# Serum prolidase activity and oxidant–antioxidant status in children with chronic hepatitis B virus infection

**DOI:** 10.1186/s13052-014-0095-1

**Published:** 2014-11-26

**Authors:** Velat Şen, Ünal Uluca, Aydın Ece, İbrahim Kaplan, Fatma Bozkurt, Fesih Aktar, Sedat Bağlı, Recep Tekin

**Affiliations:** Department of Pediatrics, Dicle University Medical School, Diyarbakir, Turkey; Department of Biochemistry, Dicle University Medical School, Diyarbakir, Turkey; Department of Infectious Diseases and Clinical Microbiology, Dicle University Medical School, Diyarbakir, Turkey

**Keywords:** Chronic hepatitis B, Prolidase, Oxidative stress, Total antioxidant capacity

## Abstract

**Background:**

Chronic hepatitis B (CHB) is a global health problem that can result in serious complications associated with collagen degradation. Prolidase is a specific imidodipeptidase that plays an important role in the breakdown of collagen. The aim of this study was to investigate prolidase activity and oxidant–antioxidant status in children with CHB.

**Methods:**

This prospective case control study includes 38 patients with CHB, 31 patients with inactive hepatitis B (IHB), and 29 healthy matched control subjects. Serum prolidase enzyme activity (SPEA), total antioxidant capacity (TAC), total oxidative activity (TOA), and malondialdehyde (MDA) level were measured and oxidative stress index (OSI) was calculated for each group.

**Results:**

Patients with CHB had significantly higher SPEA levels (207.82 ± 186.80 IU/L) than did the controls (58.6 ± 38.1 IU/L) and IHB patients (67.1 ± 39.9) (p < 0.001). CHB patients also had significantly higher TOA (45.0 ± 19.9 vs. 29.4 ± 11.7 (μmolH_2_O_2_ Eq./L), p = 0.005), OSI (33.1 ± 21.4 vs. 17.5 ± 10.2, p = 0.002) and MDA (13.4 ± 4.0 vs. 7.8 ± 2.6 μm/L, p < 0.001) values compared with the controls. TOA (32.0 ± 10.0) and OSI (15.4 ± 11.0) values of IHB patients were significantly lower than those of CHB patients (p < 0.05). SPEA had significant correlations with HBV- DNA and ALT values (r =0.514 and r =0.454, p < 0.001).

**Conclusion:**

Our results suggest that prolidase activity can be considered as a reliable marker for CHB and increased oxidative stress appears to be related to chronicity of the disease.

## Introduction

The hepatitis B virus (HBV) infection is a worldwide healthcare problem. According to the World Health Organization (WHO), approximately two billion people in the world have been infected by HBV, with more than 240 million of these having chronic hepatitis B (CHB) infection [1]. Despite the existence of effective vaccines and improvements in diagnosis and treatment, nearly one million CHB patients die every year due to complications of hepatitis B [2]. It has been reported that 3–5% of chronic carriers develop cirrhosis and 0.01–0.03% of chronic carriers develop hepatocellular carcinoma HCC before adulthood [1]. Although these complications are rare in childhood, previous studies reported that the disease can cause unfavorable future consequences [3]. Therefore, physicians should monitor all children with HBV infection, whether or not they are symptomatic, with respect to disease progression to chronicity.

A major clinical question facing the medical community today is how to better monitor children with HBV infection without using invasive techniques. Several methods have already been developed, however, none of them was validated in pediatric population. Therefore, non invasive methods that can accurately predict the severity of liver disease for patient follow up should be developed.

Prolidase is a cytosolic and multifunctional exopeptidase that possesses the unique ability to degrade iminodipeptidase, which releases carboxy-terminal proline or hydroxyproline from oligopeptides [4]. Collagen is an important substrate of prolidase due to its high contents of amino acids. It has been shown that serum prolidase enzyme activity (SPEA) is elevated in conditions that are characterized by chronic inflammation of the tissue and/or increased turnover of collagen. Previous studies have investigated prolidase activity in different clinical conditions, such as rheumatic diseases [5], asthma [6], and thalassemia [7]. However, there are few studies regarding prolidase activity in liver diseases, especially those in childhood. In one study, plasma prolidase was elevated in adults with nonalcoholic fatty liver disease [8]. In another study, patients with cirrhosis were found to have increased SPEA levels [9].

Reactive oxygen species (ROS) are produced by the metabolism of normal cells and are thought to play an important role in a variety of pathophysiological processes involving increased oxidative stress [10]. The proper balance between antioxidants and prooxidants is critical for normal function. An imbalance favoring prooxidants is defined as oxidative stress. Increased oxidative stress leads to hepatic tissue damage in liver diseases. It has been proposed that oxidative stress contributes to the progression and deterioration of damage caused by viral hepatitis [11]. Malondialdehyde (MDA) is one of the most important end product of free radical reactions. MDA has various cytotoxic effects, including enzyme inactivation and inhibition of DNA, RNA and protein synthesis, which may result in chronic viral hepatitis [12].

To our knowledge, there is no published data regarding prolidase activity in children with HBV infection. Therefore, our aim in this study was to investigate serum prolidase activity, for the first time, as a biochemical marker in children with HBV infection. In addition, we investigated oxidative stress markers, including total antioxidant capacity (TAC), total oxidant activity (TOA), oxidative stress index (OSI), and malondialdehyde (MDA), in these children.

## Materials and methods

### Patients

A total of 98 children (38 CHB, 31 inactive hepatitis B, and 29 healthy controls) who were followed up at the Pediatric Outpatient Clinics of Dicle University Hospital were included in this study. The patients were recruited prospectively between January 2014 and April 2014. The control group consisted of children who were undergoing routine checkups or presurgical examination for elective minor surgery, such as inguinal hernia repair or circumcision. The children in the control group had normal physical examinations and blood biochemistry and their medical history gave no findings of systemic illnesses or recent disease. None of these children had positive hepatitis B serum markers.

The diagnosis of CHB infection was based on the following: persistent or intermittently elevated liver enzymes, serum alanine aminotransferase (ALT) greater than 1.5 times the normal value (less than 40 IU/L), a positive hepatitis B surface antigen (HBsAg) test result for more than 6 months, and serum HBV DNA >2.000 IU/ml. Patients were included in the study as inactive HBsAg carriers (inactive hepatitis B, IHB) if they had the following: a positive HBsAg longer than 6 months, a negative Hepatitis B “e” antigen (HBeAg), serum HBV DNA <2.000 IU/ml, normal ALT and aspartate aminotransferase (AST) levels, and negative anti-HDV during their follow-up period [13].

Patients underwent regular follow-ups and were selected for assessment of liver function based on prolidase activity and oxidative status. A detailed medical history and clinical examination were performed in all patients. They also underwent evaluation for the existence of diseases other than CHB on presentation. Anthropometric characteristics, including weight, height and body mass index (BMI) z-scores were determined and recorded.

### Exclusion criteria

Patients were excluded from the study if they met the following criteria: aged less than 3 years or more than 17 years; had concomitant diseases that could affect liver functions or potentially lead to liver damage, such as autoimmune hepatitis, α1-antitrypsin deficiency and Wilson disease; diagnosed with malignancy; diagnosed with hepatitis D and hepatitis C infections; or received hepatotoxic drugs. Patients were also excluded if they had a history of suggestive trauma or fracture, systemic or local infections, juvenile rheumatoid arthritis, findings in favor of other liver and connective tissue disorders, were receiving corticosteroids and non-steroid anti-inflammatory drugs capable of interfering with free radical production or SPEA, and liver and kidney failure. In addition, patients with liver cirrhosis, irrespective of the etiology, were also excluded.

### Biochemical analyses

Patients were advised not to take any medications for 24 hours prior to blood sampling. Blood samples were withdrawn from an antecubital vein, preferably after an overnight fast, and were immediately centrifuged at 4,000 rpm at 4°C for 10 min and then transferred into Eppendorf tubes. Samples were transferred on ice and stored at −80°C until further analysis.

Baseline laboratory results of all patients were obtained on admission and measured by an enzymatic colorimetric method by an Abbot ARCHITECT *C*16000 (Illinois, USA) instrument in the Biochemistry Laboratory. These values included serum glucose, albumin, ALT, AST, gamma glutamyl transferase (GGT) and total bilirubin (TBIL). Additionally, Prothrombin Time (PT) and activated Partial Thromboplastin Time (aPTT) were measured and complete blood counts were performed. Hepatobiliary ultrasound was performed for all patients.

#### Measurement of serum prolidase activity

SPEA was determined using a commercially available quantitative enzyme-linked immune sorbent assay (ELISA) technique (Hangzhou Eastbiopharm Company, Hangzhou, China) according to the manufacturer’s instructions.

#### Measurement of total antioxidant capacity

TAC of the supernatant fractions was determined using a novel automated measurement method developed by Erel [14]. In this method, the antioxidative effect of the sample against potent-free radical reactions (initiated by a hydroxyl radical) is measured. The results are expressed as μmol Trolox eq./L.

#### Measurement of total oxidant activity

TOA of supernatant fractions was determined using a novel automated measurement method developed by Erel [15]. The assay is calibrated with hydrogen peroxide and the results are expressed as μmol H_2_O_2_ equiv./L.

#### Oxidative stress index

OSI indicates the degree of oxidative stress, and is calculated as follows: OSI (arbitrary units) = [TOA/TAC] × 100^14^.

#### Lipid oxidation determination

MDA levels in plasma were measured via high-pressure liquid chromatography (HPLC) based on differentiation with dinitrophenylhydrazine [16]. The MDA results are expressed as μmol/L.

Our study was conducted in accordance with the Declaration of Helsinki and approved by the Institutional Review Board of Dicle University Medical School. Written informed consent was obtained from each participant and/or from his or her legal caregivers prior to enrolling in the study.

### Statistical analysis

Results were expressed as means and standard deviations (SD). A one-sample Kolmogorov-Smirnov test was used to determine if the data was normally distributed. Intergroup comparisons were performed, based on data distribution pattern, using one-way ANOVA or Kruskal-Wallis test for three independent groups and Student’s *t*-test or Mann–Whitney *U*-test for two independent groups. Relationships between variables were determined using the Spearman correlation analysis. P values less than 0.05 were considered statistically significant. All data were analyzed using the statistical package SPSS 18.0 for Windows (IBM Corporation, Armonk, NY).

## Results

A total of 38 children with CHB (27 boys, 11 girls), 31 with IHB (21 boys, 10 girls), and 29 matched healthy control subjects (14 boys, 15 girls) were enrolled in this study between January 2014 and April 2014. The demographic and biochemical characteristics of the study groups are listed in Table 1. The mean age and age ranges of the groups were as follows: CHB group, 11.36 ± 3.60 years (range, 3–17); IHB group, 12.22 ± 2.70 years (range, 6–16); and the control subjects, 11.31 ± 2.73 years (range, 7–16). There were no significant differences between the groups in terms of age, gender distribution and anthropometric measurements (p > 0.05, for all) (Table 1).

**Table 1:** **Demographic and biochemical characteristics of the study groups**

	**Chronic hepatitis B** ^**(a)**^ **(n = 38)**	**Inactive hepatitis B** ^**(b)**^ **(n = 31)**	**Controls** ^**(c)**^ **(n = 29)**	**p**
Age, years	11.4 ± 3.6	12.2 ± 2.7	11.3 ± 2.7	^a-b-c^NS
BMI, z-scores	−0.156 ± 1.011	−0.134 ± 0.998	0.240 ± 0.698	^a-b-c^NS
Serum glucose (mg/d L)	93.6 ± 9.1	93.0 ± 11.7	96.4 ± 14.5	^a-b-c^NS
Serum ALB (g/dL)	3.0 ± 0.3	4.0 ± 0.3	4.0 ± 0.2	^a-b-c^NS
TBIL (mg/dL)	0.65 ± 0.93	0.59 ± 0.39	0.41 ± 0.16	^a-b-c^NS
ALT (IU/L)	73.6 ± 64.8^*^	28.5 ± 12.9	24.9 ± 6.6	^a-b^ < 0.001, ^a-c^ < 0.001, ^b-c^NS, ^a-b-c^ < 0.001
AST (IU/L)	59.9 ± 53.6	27.8 ± 8.3	25.9 ± 5.8	^a-b^ < 0.001, ^a-c^ < 0.001, ^b-c^NS, ^a-b-c^ < 0.001
GGT (IU/L)	35.9 ± 47.8	18.5 ± 9.9	18.9 ± 6.1	^a-b-c^NS
aPTT (second)	28.58 ± 3.23	31.10 ± 11.46	34.58 ± 15.72	^a-b-c^NS
INR	1.09 ± 0.07	1.06 ± 0.08	1.05 ± 0.06	^a-b-c^NS

Data presented as mean ± standard deviation, ^*^ *p* < 0.001vs. inactive hepatitis B and controls.

BMI, body mass index; ALB, albumin; TBIL, total bilirubin, ALT: Alanine aminotransferase, AST: Aspartate aminotransferase, GGT: Gamma glutamyl transferase, INR: International normalized ratio of prothrombin time; and aPTT: Activated partial thromboplastin time, NS: Not significant.

The serum glucose, albumin, total protein, total bilirubin, and direct bilirubin values of three groups were within normal limits, and there were no significant differences between them (p > 0.05, for all) (Table 1). In addition, there were no significant differences between the PT and aPTT values in children with HBV infection and the controls (p = 0.077 and p = 0.758, respectively) (Table 1).

In the CHB group, serum ALT levels ranged from 6 to 274 IU/L, and 22 (57.9%) of the children in this group had high (>40 IU/L) ALT values. In addition, 16 (42.1%) children in the CHB group had high (>40 IU/L) AST (range, 15–230 IU/L) values; and seven (18.4%) of them had high (>29 IU/L) GGT (range, 8–180 IU/L) levels. Seven (22.6%) of the IHB patients had transient mild ALT elevations (range, 6–59 IU/L, ALT 40–50 IU/L in 5 patients, 50–59 IU/L in two patients). One (3.2%) of IHB patients had transiently elevated AST (range, 16–48 IU/L), and GGT (range, 7–53 IU/L) values. During follow-up elevated ALT, AST and GGT values of IHB patients returned to normal values. Children with CHB had significantly higher ALT and AST levels than those in IHB and the control groups (p < 0.001 for each) (Table 1).

The HBV DNA values in the CHB group ranged from 12×10^3^ to 170×10^6^ IU/mL, the HBsAg values ranged from 174 to 5385 IU/mL (positive in all patients), and 3 (7.9%) of these patients had positive AntiHBe. The HBV DNA values in the IHB group ranged from 100 to 1440 IU/mL, the HBsAg ranged from 100 to 6277 IU/mL, and 1 (3.3%) of these patients had positive HBeAg. CHB patients had significantly higher HBV DNA and HBsAg titers than did the IHB patients (p < 0.001, for each comparison).

Table 2 summarizes the prolidase activity and serum markers of oxidative stress (TAC, TOA, OSI and MDA) in the three groups. Significantly higher SPEA was found in CHB patients compared with the IHB patients and the control subjects (p < 0.001, for each) (Table 2). However, no significant difference was found in SPEA values between the IHB subjects and the healthy controls (p > 0.05) (Table 2).

**Table 2:** **Prolidase activity and oxidative–antioxidative status in the study groups (mean ± standard deviation)**

	**Chronic hepatitis B (n = 38)**	**Inactive hepatitis B (n = 31)**	**Controls (n = 29)**	^*^ **p**
SPEA (IU/L)	207.8 ± 186.8^a,e^	67.1 ± 39.9	58.6 ± 38.1	<0.001
MDA (μm/L)	13.3 ± 4.0^a,h^	7.8 ± 3.6	7.8 ± 2.6	<0.001
TAC (μmol Trolox Eq t/l)	1.77 ± 0.90^b^	2.28 ± 0.88	2.46 ± 0.67	0.015
TOA (μmolH_2_O_2_ Eq./L)	45.0 ± 19.9^c,f^	32.0 ± 10.0	29.4 ± 11.7	0.001
OSI (H_2_O_2_/Trolox)	33.1 ± 21.4^d,g^	15.4 ± 11.0	17.5 ± 10.2	<0.001

CHB: Chronic Hepatitis B, IHB: Inactive Hepatitis B, SPEA: Serum prolidase enzyme activity, TAC: Total antioxidant capacity, TOA: Total oxidant activity, OSI (Arbitrary Unite): Oxidative stress index, and MDA: Malondialdehyde.

^*^Difference between three groups with Kruskal-Wallis test.

Differences between pairwise groups with Mann–Whitney *U* test:

^a^Compared with group control (p < 0.001).

^b^Compared with group control (p = 0.004).

^c^Compared with group control (p = 0.005).

^d^Compared with group control (p = 0.002).

^e^Compared with group IHB (p < 0.001).

^f^Compared with group IHB (p = 0.001).

^g^Compared with group IHB (p < 0.001).

^h^Compared with group IHB (p < 0.001).

The CHB patients had significantly higher plasma MDA, TOA, and OSI levels compared with the IHB patients and the control subjects (p < 0.05) (Table 2). The TAC levels of the CHB patients were significantly lower than those of the healthy controls (p = 0.004). However, no significant differences were found in MDA, TAC, TOA and OSI levels between CHB and IHB groups (p > 0.05) (Table 2).

**Figure 1:**
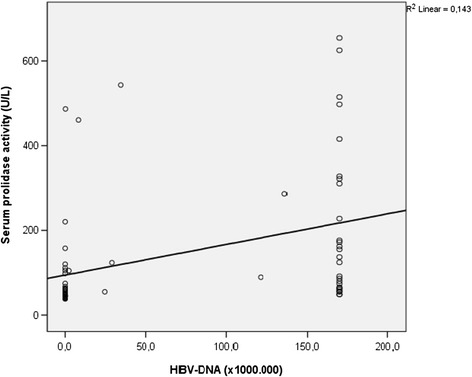
**The relationship between serum prolidase activity and HBV-DNA (r = 0.514, p < 0.001).**

**Figure 2:**
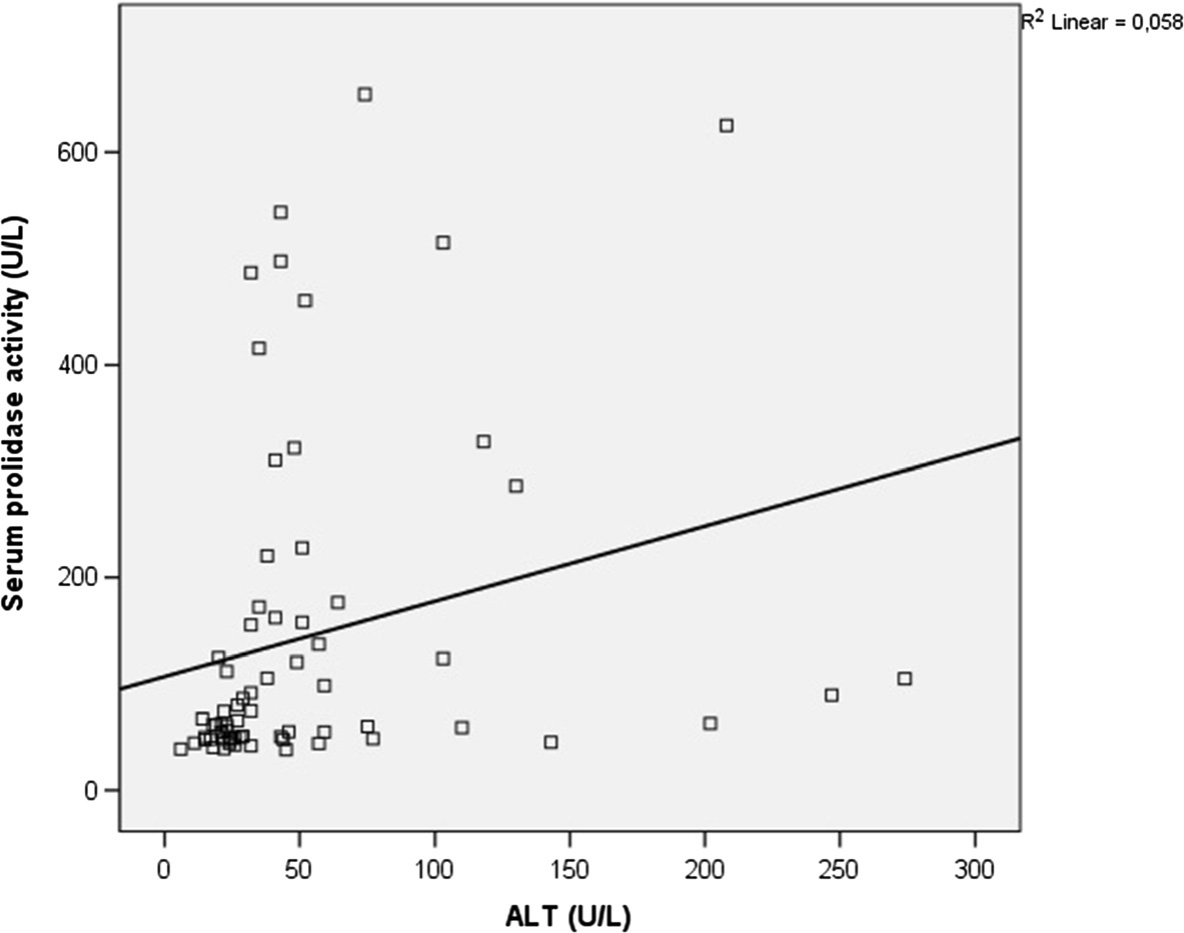
**The relationship between serum prolidase activity and alanine aminotransferase (ALT) (r = 0.454, p < 0.001).**

**Figure 3:**
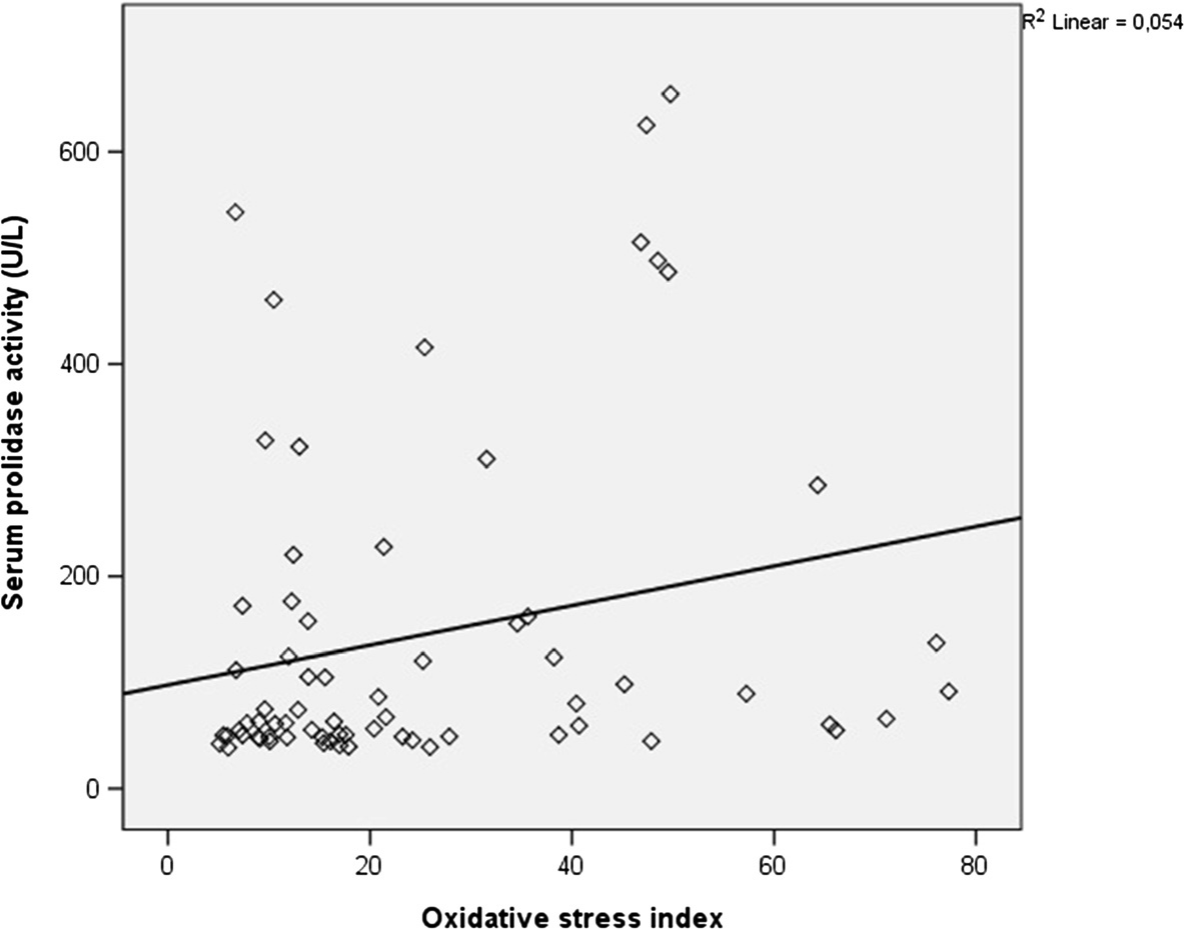
**The relationship between serum prolidase activity and oxidative stress index (r = 0.267, p = 0.027).**

**Figure 4:**
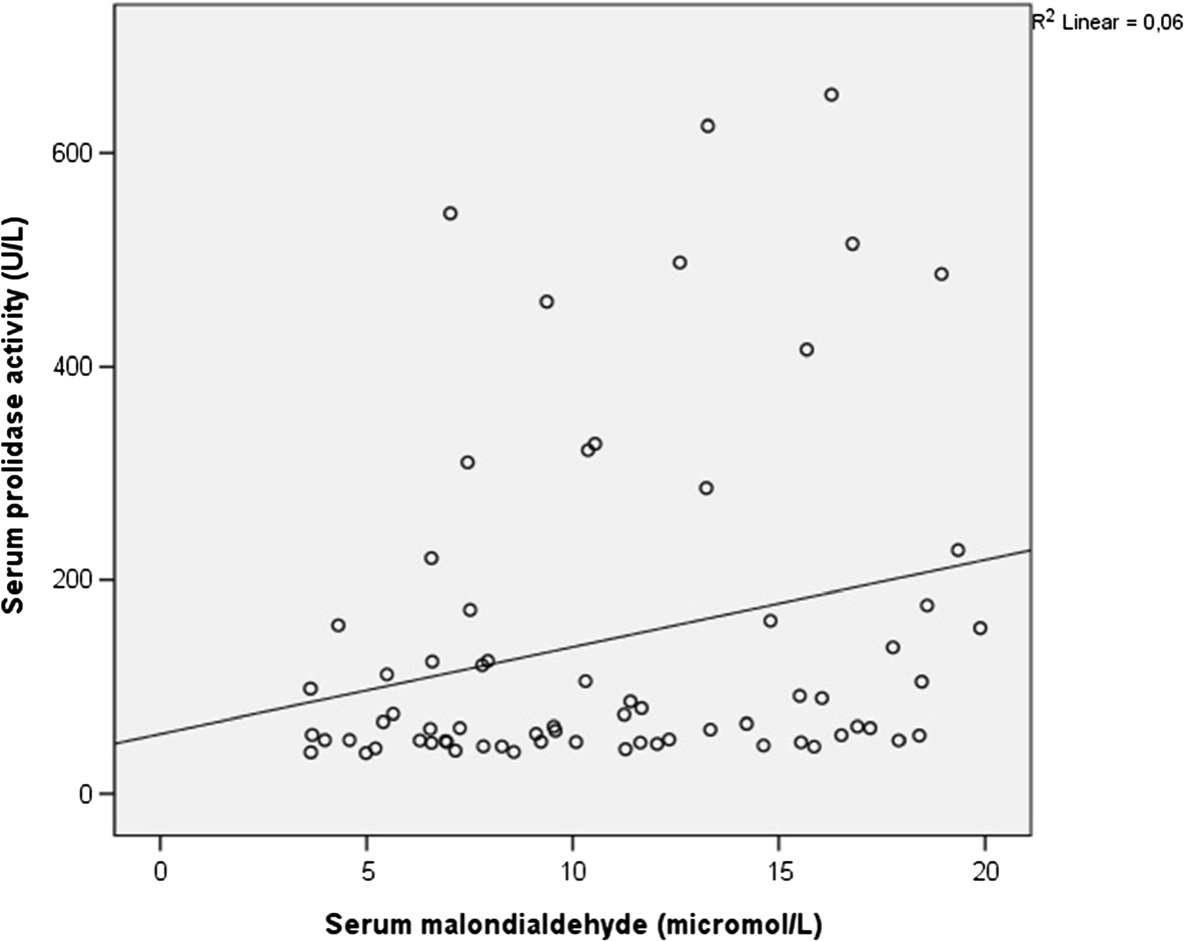
**The relationship between serum prolidase activity and malondialdehyde (r = 0.281, p = 0.020).**

There was a positive significant correlation between prolidase activity and HBV- DNA values (*r* =0.514, p < 0.001) in total CHB and IHB patients (n = 69) (Figure 1). There was also a significant positive correlation between SPEA and ALT values (*r* =0.454, p < 0.001) while CHB and IHB groups were taken as a whole group (n = 69) (Figure 2). Serum prolidase activity had weak positively correlations with MDA and OSI (*r* =0.281, *r* =0.267, p < 0.05) (Figures 3 and 4). However, no significant correlations were found between SPEA and TAC or between SPEA and TOA (p > 0.05 and p = 0.050, respectively) (n = 69).

HBV-DNA was positively correlated with MDA, TOA and OSI levels (*r* = 0.557, *r* = 0.417, *r* = 0.497, p < 0.001 for each), and negatively correlated with TAC levels (*r* = −0.246, p = 0.042). Significant positive correlations were found between ALT and MDA levels as well as between TOA and OSI values (*r* =0.270, *r* =0.263, *r* =0.290, p < 0.05).

There were no significant correlations between SPEA and patient age or hematological variables, including hemoglobin, hematocrit, red blood cell count, and platelet counts (data not shown, p > 0.05).

## Discussion

Hepatitis B virus (HBV) infection is an important disease worldwide and appropriately monitoring of these patients is important [17]. Although liver biopsy remains gold standard for determining disease progression, it is not practical to perform repeated liver biopsies for all children who require long-term assessments. Therefore, diagnostic and prognostic noninvasive markers are more appropriate for practical assessments.

To our knowledge, the current study is the first to demonstrate significantly increased serum SPEA levels in children with CHB compared with IHB patients and the healthy controls. The present study is also the first to evaluate both oxidative status and prolidase activity in CHB and IHB children. We found increased prolidase enzyme activity and oxidative stress, and decreased antioxidant levels in children with CHB.

The mechanisms of the progression from inactive disease state to chronicity are not clearly understood. Collagen fibrils act as a frame to support the hepatocytes and impairment of this structure results in fibrosis [18]. In previous studies, the level of fibrosis has been evaluated with non-invasive biochemical tests, including N-terminal propeptide of type III collagen, FibroTest, SteatoTest, and NashTest [19]. However, some of these tests are not widely available, and elevated test results have been found also in several non-specific conditions.

The prolidase enzyme is present in various tissues and plasma and its physiological role in humans is well-understood. During protein catabolism, prolidase catalyzes the degradation of intracellular collagen, and its activity may be correlated with the rate of collagen degradation [20]. It is believed that its existence in a wide variety of tissues and changes in prolidase activity may play important roles in extracellular matrix turnover and the development and outcome of several diseases [21,22]. To our knowledge, until the current study, there is no report concerning prolidase activity in pediatric CHB patients.

An increase or decrease in prolidase activity can demonstrate the existence of a disease state as well as the progression of the condition. Although some authors suggest that prolidase activity decreases in some disease conditions, such as asthma [6] and chronic obstructive pulmonary disease [23], increased prolidase activity has been reported in some other diseases and cancers [7,24]. SPEA has been investigated in patients with liver damage, such as chronic liver diseases [25], alcoholic liver disease [26], and chronic hepatitis C (CHC) infection [27]. One study investigated SPEA in patients with nonalcoholic steatohepatitis (NASH), and found significantly elevated prolidase enzyme activity in NASH than simple steatosis. In addition, some studies have reported a significant correlation between SPEA and stage of liver fibrosis [8].

In and adult study, prolidase levels were found to be higher in CHB and IHB compared to the control group and no correlation of prolidase activity was found with fibrosis, and histological activity index [28]. Our study included pediatric patients, and therefore, it is different from previously published studies. Our study evaluated prolidase activity in CHB and IHB children. We found higher prolidase activity in CHB children than IHB and the healthy control group. However, as a limitation of our study, we did not perform liver histology.

There were significant positive correlations between the prolidase activity, HBV- DNA, and ALT levels in our study groups. These correlations may indicate the reliability of serum prolidase measurements and the parallel changes of these enzyme activities in children with CHB. The positive correlations that we found between prolidase activity and markers of oxidative stress includeing MDA, TOA, and OSI levels are consistent with the hypothesis that both serum prolidase activity and oxidative parameters indicate the impairment of hepatic functions [27]. Increased MDA, TOA, and OSI levels and decreased TAC levels in our study, support the hypothesis that increased oxidative stress may contribute to the pathogenesis of CHB and the exacerbation of the disease. Indeed, it has been demonstrated that the amount of ROS in healthy human liver was significantly lower than found in livers affected by hepatitis B [29]. Recently, there has been increasing body of evidence proposing that oxidative stress may play a critical role in various liver diseases, including chronic hepatitis [30]. Our results, which indicate decreased antioxidant capacity and increased levels of some important oxidants, such as MDA, are in accordance with the results of other investigators [10,12].

One potential limitation of our study was its cross-sectional design. However, this is a preliminary study, which provided information regarding collagen metabolism and oxidative stress in children with CHB by evaluating serum prolidase activity and by determining TAC, TOA, OSI and MDA.

In conclusion, this study provides novel clinical findings, including a relationship between increased SPEA and the presence of CHB in children. This may be interpreted as evidence of increased collagen turnover in CHB. Monitoring serum prolidase activity in clinical practice may be a suitable and useful method for evaluating hepatitis B advance to chronicity in children. Further longitudinal and prospective studies are needed to clarify the pathophysiological role of serum prolidase activity in the progression of the disease process.
